# Effects of Endothelial Progenitor Cell-Derived Microvesicles on Hypoxia/Reoxygenation-Induced Endothelial Dysfunction and Apoptosis

**DOI:** 10.1155/2013/572729

**Published:** 2013-10-28

**Authors:** Jinju Wang, Shuzhen Chen, Xiaotang Ma, Chuanfang Cheng, Xiang Xiao, Ji Chen, Shiming Liu, Bin Zhao, Yanfang Chen

**Affiliations:** ^1^Department of Pharmacology & Toxicology, Boonshoft School of Medicine, Wright State University, 3640 Colonel Glenn Highway, Dayton, OH 45435, USA; ^2^Institute of Neurology, Affiliated Hospital of Guangdong Medical College, Zhanjiang 524001, China; ^3^Cardiovascular Department, The Second Hospital Affiliated to Guangzhou Medical University, Guangzhou Institute of Cardiovascular Disease, Guangzhou 510000, China

## Abstract

Oxidative stress-induced endothelial dysfunction plays a key role in ischemia/reperfusion injury. Recent evidence indicates that endothelial progenitor cell-derived microvesicles (EPC-MVs) can promote angiogenesis of endothelial cells (ECs). Here, we investigated the potential effects of EPC-MVs on hypoxia/reoxygenation (H/R) injury in human brain microvascular ECs (hb-ECs). MVs were prepared from EPCs cultured in a serum deprivation (SD) medium (starving stress, sEPC-MVs) or SD medium containing tumor necrosis factor-**α** (TNF**α**) (apoptotic stress, aEPC-MVs). H/R injury model of hb-ECs was produced by 6 hr hypoxia (1% O_2_) and 24 hr reoxygenation. The H/R hb-ECs were co-cultured with EPC-MVs. Results showed that (1) H/R hb-ECs were dysfunctional and coupled with increased apoptosis and ROS overproduction; (2) under two different conditions, EPCs displayed remarkable difference in caspase 3 and miR126 expression, which were carried by the corresponsive EPC-MVs; (3) functionally, sEPC-MVs had beneficial effects on H/R hb-ECs, whereas aEPC-MVs had detrimental effects; (4) the diverse effects of sEPC-MVs and aEPC-MVs were associated with the changes in miR126 and eNOS expression and were abolished by PI3K inhibitor. In conclusion, sEPCs-MVs and aEPC-MVs are functionally different on hb-EC apoptosis and dysfunction via their carried RNAs associated with ROS production and PI3K/eNOS/NO pathway.

## 1. Introduction

Reactive oxygen species (ROS) are well known to mediate ischemia/reperfusion (I/R) injury [1]. ROS-induced vascular endothelial cell (EC) injury is the first key step in the pathogenesis of ischemia/reperfusion injury. Therefore, attenuating oxidative stress-induced EC injury could be an important strategy in the management of I/R injury on tissues. Several strategies for inhibiting ROS production or increasing their degradation and scavenging have been attempted and turned out to be less effective [2]. Therefore, novel approaches are greatly demanded for preventing I/R injury. 

Endothelial progenitor cells (EPCs) are circulating bone marrow-derived precursors which are able to exert a protective effect in experimental models of hindlimb ischemia [3] and myocardial infarction [4]. Previous studies have shown that EPCs recruited into the kidney could induce tissue repair via secretion of proangiogenic factors [5, 6]. 

Microvesicles (MVs) are small particles 0.1–1 *μ*m in size, which are shed from the plasma membrane of various cell types [7, 8]. They carry proteins and gene messages (mRNAs and miRNAs). Because of their ability to merge with target cells, MVs can deliver their contents into the cells they communicate with [8–10]. Recent studies have reported that MVs produced from mesenchymal stem cells and EPCs could exert protective effects in experimental models of acute/chronic kidney injury [11–13]. In addition, another study demonstrates that MVs released from EPCs (EPC-MVs) can trigger angiogenesis by a horizontal transfer of mRNA [14]. Nevertheless, there is little information regarding the effects of EPC-MVs on ECs injured by hypoxia/reoxygenation (H/R).

In this study, we aimed to investigate the potential effects of EPC-MVs on human brain microvascular ECs (hb-ECs) injured by H/R and the underlying mechanisms.

## 2. Material and Methods

### 2.1. Culture and Characterization of EPCs

The mononuclear cells (MNCs) isolated from mouse bone marrow were used for EPC culture as previously described [15, 16]. In brief, the cells were counted and plated (1 × 10^6^ cells/well) on fibronectin-coated 24-well plates (BD Bioscience, San Jose, CA) and then grown in endothelial cell basal medium-2 (EBM-2) supplemented with 5% FBS containing EPC growth cytokine cocktail (Lonza, Walkersville, MD). After 3 days, nonadherent cells were removed by washing with PBS. Thereafter, culture medium was changed every 2 days. Cultured cells were characterized by double-positive Di-LDL and Bs-Lectin staining as previously reported [15, 16].

### 2.2. Preparation of EPC-MVs

EPC-MVs were prepared from EPCs using two different stimulations, starving stress and apoptotic stress, based on previous reports with slight modifications [13, 17]. EPCs were cultured in serum deprivation (SD) medium for 24 hrs for generating starving stress EPC-MVs (sEPC-MVs). For generating apoptotic stress EPC-MVs (aEPC-MVs), EPCs were cultured in SD medium supplemented with 25 ng/mL TNF*α* (R&D systems, MN) for 24 hrs. MVs collected from EPCs cultured in EBM-2 served as a control (control MVs).

### 2.3. Characterization and Labeling of EPC-MVs

EPC-MVs were collected from EPC modified culture medium as previously described with slight modification [13]. In brief, EPC culture medium was collected, centrifuged at 2000 g for 20 mins to remove cells and debris, and ultracentrifuged at 120,000 g for 1 hr. The protein concentration of EPC-MV preparations was quantified by the Bradford method (Bio-Rad, Hercules, USA). A concentration of 10 *μ*g/mL EPC-MVs was used for coculture experiments. In selected experiments, EPC-MVs were pretreated with 0.1% Triton-100 for 5 mins, then treated with 200 u/mL RNase (Qiagen, CA) for 90 mins at 37°C, washed, and pelleted by ultracentrifugation. To verify the effect of RNase, the total RNAs of EPC-MVs were extracted using the RNA isolation kit (Ambion, NY), and the RNA concentration was determined using quantitative assay (Thermo Scientific, Nanodrop 2000c, FL). For flow cytometry analysis [18], EPC-MVs were resuspended and incubated for 30 mins at 4°C in the dark with Alexa-488-labeled Annexin V, PE-conjugated VEGFR2, or FITC-conjugated CD34, 5 *μ*L, respectively. Isotype matched (IgG) nonspecific antibodies served as negative controls. All antibodies were purchased from eBioscience (San Diego, CA). After incubation, labeled cells were resuspended with PBS and analyzed under flow cytometry (Accuri C6 flow cytometer, Ann Arbor, MI). For TEM analysis [18], EPC-MVs were fixed with 2% glutaraldehyde and postfixed with 1% osmium (all were purchased from electron microscopy science, Hatfield, PA), then embedded with Spurr resin (Sigma, Louis, MO), and baked at 60°C according to the manufacturer's instruction. Ultrathin sections (60–80 nm) were prepared with MT7000, mounted on 300-mesh copper grids, and stained with uranyl acetate and lead citrate. All samples were examined with an EM 208 (Philips) transmission electron microscope at an accelerating voltage of 70 KV.

EPC-MVs were labeled with PKH26 (Sigma Aldrich, St. Louis, MO) according to the manufacturer's protocol with some modifications [19]. In brief, EPC-MVs were labeled with 2 *μ*M PKH26 dye in PBS for 5 mins at room temperature (RT). An equal volume of FBS was added to stop staining. EPC-MVs were then ultracentrifuged and resuspended with culture medium for coculture experiments.

### 2.4. H/R Injury Model in Hb-ECs

Hb-ECs were purchased from Cell Systems (Kirkland, WA) and cultured according to the manufacturer's protocol. Briefly, cells were cultured in CSC complete medium containing 10% serum, 2% human recombinant growth factors, and 0.2% antibiotic solution under standard cell culture conditions (5% CO_2_, 37°C). All medium and supplement reagents were purchased from Cell Systems. Medium was changed twice a week. Passages 4 to 13 of hb-ECs were used for experiments in this study. For producing an H/R injury model on hb-ECs, hb-ECs were cultured as previously described with slight modifications [20]. Briefly, the hb-ECs were changed with fresh culture medium and cultured for 6 hrs in a hypoxic chamber (1% O_2_, 5% CO_2_, and 94% N_2_; Biospherix hypoxia chamber, NY); cells were then reoxygenated by incubation in a standard 5% CO_2_ incubator for 24 hrs. Some cells were harvested for apoptotic assay, western blot, or quantitative real-time PCR (qRT-PCR) analysis. Some plates of cells were pretreated with or without LY294002 (PI3K inhibitor) and then cocultured with sEPC-MVs, aEPC-MVs, RNase treated sEPC-MVs (RNase-sEPC-MVs), or RNase treated aEPC-MVs (RNase-aEPC-MVs). All experiments were repeated four times. At least six plates per experiment were used in each group.

### 2.5. Coculture Assay of EPC-MVs and Hb-ECs

For coculture experiment between hb-ECs and EPC-MVs [21], the labeled EPC-MVs were resuspended with CSC medium, then added to hb-ECs, and cultured for 2 hrs in an incubator (37°C, 5% CO_2_). Cell nuclei were stained with DAPI. The interaction between EPC-MVs and hb-ECs was examined under fluorescence microscope.

### 2.6. Gene Expression Analysis

MicroRNA 126 (miR126) from EPCs, EPC-MVs, and H/R hb-ECs was extracted by using mirVana miRNA isolation kit (Ambion) following manufacturer's instructions. cDNA was synthesized using miScript reverse transcription kit (QIAGEN). Quantitative real-time PCR was conducted with miR126 specific primers and miScript SYBR Green PCR Kit (QIAGEN) on a real-time PCR system (Bio-Rad). Small nuclear RNA U6 (U6) was used as an internal control. Relative expression of miR126 was calculated using the 2^−ΔΔCT^ method [22].

### 2.7. Cell Viability Analysis

The cell viability of H/R hb-ECs was examined using methyl thiazolyl tetrazolium (MTT, Invitrogen, NY) method [23]. In brief, cells cultured in 96-well plate were incubated with 10 *μ*L MTT solution (12 mM) for 4 hrs at 37°C, then 100 *μ*L sodium dodecyl sulfate (SDS)-HCl solution was added to each well, and the cells were incubated for another 4 hrs at 37°C. The absorbance of cells was read at 535 nm. The percent of cell viability was defined as the relative absorbance of treated cells versus untreated cells.

### 2.8. Apoptosis Assay

The apoptosis assay of EPCs and H/R hb-ECs was conducted using FITC Annexin V apoptosis detection kit (BD Biosciences, CA) as previously described [23]. Briefly, cells were washed with PBS, resuspended with 100 *μ*L 1x annexin-binding buffer, incubated with 5 *μ*L FITC-conjugated Annexin V and 5 *μ*L propidium iodide (PI) for 15 mins in the dark, and then analyzed by flow cytometry. The apoptotic cells were defined as Annexin V+/PI− cells. The experiment was repeated four times. At least six plates per experiment were used in each group.

### 2.9. Tube Formation Assay

The tube formation ability of EPCs and hb-ECs cocultured with EPC-MVs was evaluated using tube formation assay kit (Chemicon) as we previously described [24]. Briefly, ECMatrix solution was thawed on ice overnight, mixed with 10x ECMatrix diluent buffer, and placed in a 96-well tissue culture plate at 37°C for 1 hr to allow the matrix solution to solidify. Then the cells were re-plated (1 × 10^4^ cells/well) onto the surface of the solidified ECMatrix and incubated for 24 hrs at 37°C. Tube formation was evaluated with an inverted light microscope and defined as a tube structure exhibiting a length 4 times its width. Five independent fields were assessed for each well, and the average number of tubes per field (magnification, 200x) was determined.

### 2.10. Measurement of ROS

Intracellular ROS production was determined by dihydroethidium (DHE) (Sigma) staining followed by flow cytometric analysis. Briefly, cells were incubated with 2 *μ*M DHE solution at 37°C for 2 hrs, washed with PBS twice, trypsinized, and centrifuged. The fluorescence intensity of cells was analyzed by flow cytometry.

### 2.11. Measurement of NO

The membrane-permeable indicator diaminofluorescein (DAF-FM) diacetate (Invitrogen, Grand Island, NY) was used to assess NO production [16]. Briefly, the hb-ECs were incubated with 2 *μ*M DAF-FM diacetate in serum-free CSC medium at 37°C for 30 mins, washed with PBS twice, then incubated with CSC medium for 20 mins to allow complete de-esterification of the intracellular diacetates. DAF-FM fluorescence was measured using a spectrofluorometer.

### 2.12. Western Blot Analysis

Proteins from H/R hb-ECs were extracted with lysis buffer. Protein lysates were electrophoresed through SDS-PAGE gel and transferred onto PVDF membranes. The membranes were blocked for 1 hr and incubated with primary antibodies against eNOS (Cell Signaling Technology) and *β*-actin (Sigma) at 4°C overnight. After washing 3 times, membranes were incubated with horseradish-peroxidase- (HRP-) conjugated IgG (Jackson ImmunoResearch Lab) for 1 hr at RT. Blots were then developed with enhanced chemiluminescence developing solutions and quantified under ImageJ software.

### 2.13. Enzyme-Linked Immunosorbent Assay

The levels of caspase 3 in EPCs, EPC-MVs, and H/R hb-ECs were measured by enzyme-linked immunosorbent assay (ELISA) [25]. Briefly, cells or EPC-MVs were washed in PBS and their extracts were prepared according to the manufacturer's instructions before being analyzed by ELISA (R&D System, Quantikine).

### 2.14. Statistical Analysis

All data was expressed as mean ± SEM. Comparison for two groups was examined by Student's *t*-test. Multiple comparisons were performed by one- or two-way ANOVA. SPSS software version 17.0 was used. For all tests, a value of *P* < 0.05 was considered statistically significant.

## 3. Results

### 3.1. The sEPC-MVs and aEPC-MVs Carried the Characters of Their Parent EPCs

**Figure 1:**
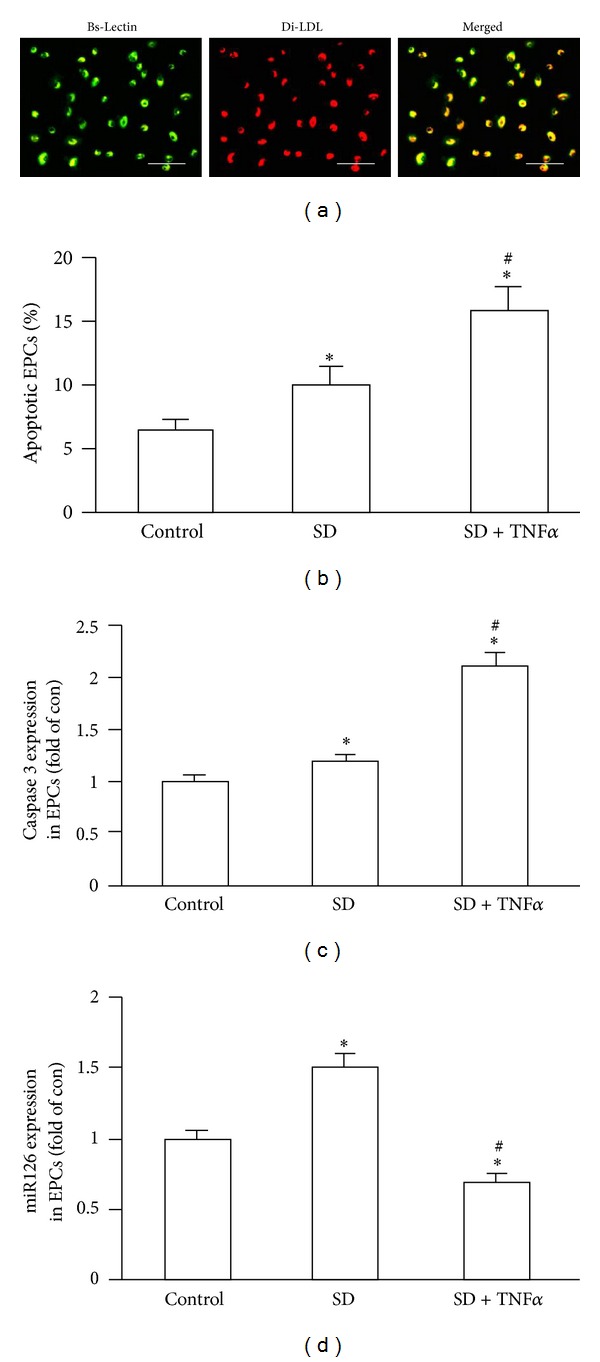
Effects of serum deprivation (SD) alone and SD plus TNF*α* on EPC apoptosis, caspase 3, and miR126 expression. (a) Representative images showing EPC characterization of Bs-Lectin and Di-LDL double staining. Scale bar: 100 *μ*m. (b) Apoptosis (Annexin V+PI−) of stimulated EPCs. (c) Caspase 3 expression in stimulated EPCs. (d) MiR126 expression in stimulated EPCs. *P < 0.05, versus control; ^#^ *P* < 0.05, versus SD; *N* = 4/group.

As we previously reported [16], EPCs were defined as the cells uptaking Di-LDL and binding with Bs-Lectin (Figure 1(a)). As expected, EPCs in an apoptotic stress condition (SD plus TNF*α* stimulation) had a significantly higher apoptotic rate than that of EPCs in a starving stress (SD only) or normal culture condition (versus control or SD; *P* < 0.05; *n* = 4/group; Figure 1(b)). Moreover, gene expression of caspase 3 was upregulated, whereas miR126 was down-regulated in EPCs in the SD + TNF*α* group as compared to control group. In SD group, both caspase 3 and miR126 expressions were higher than those in control (versus control; *P* < 0.05; *n* = 4/group; Figures 1(c)-1(d)). 

**Figure 2:**
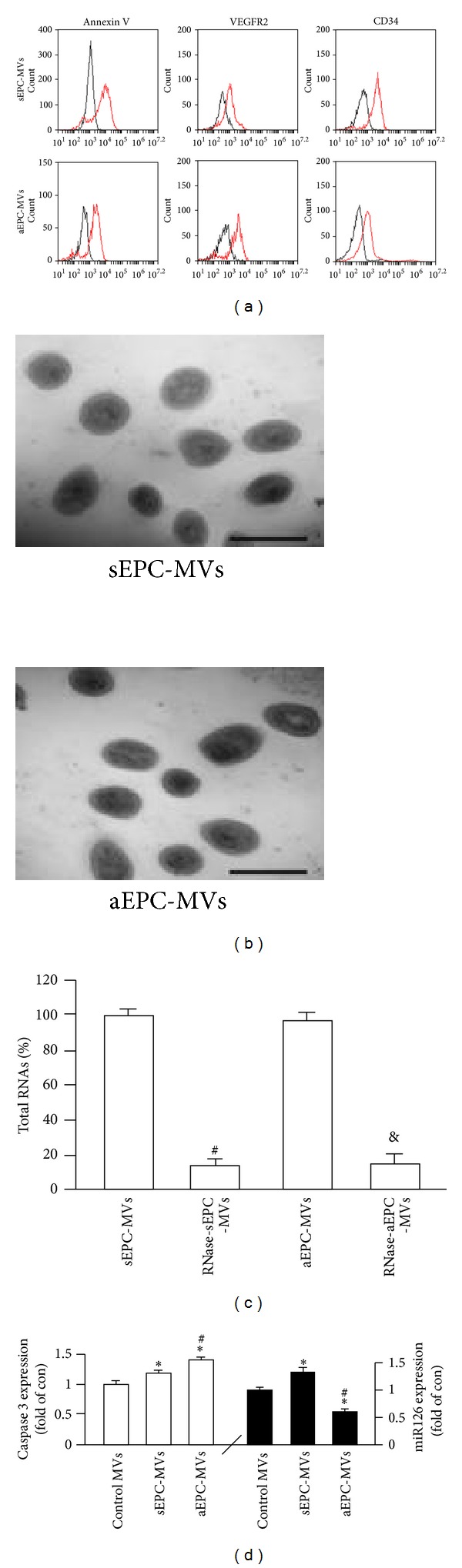
EPC-MV characterization, modification, caspase 3 and miR126 expression. (a) Flow cytometric plots showing Annexin V, CD34 and VEGFR2 expressions (isotype controls: left curves; antibodies: right curves) in EPC-MVs. (b) TEM image showing similar spherical morphology of sEPC-MVs and aEPC-MVs. Scale bar: 500 nm. (c) Summarized data showing effective digestion of EPC-MVs total RNAs by RNase treatment. (d) Caspase 3 and miR126 expression in control MVs (generated from basal condition), sEPC-MVs, and aEPC-MVs. *P < 0.05, versus control; ^#^ *P* < 0.05, versus sEPC-MVs; ^&^ *P* < 0.05, versus aEPC-MVs; *N* = 4/group. TEM and transmission electron microscopy.

According to the flow cytometric analysis, both aEPC-MVs and sEPC-MVs positively expressed Annexin V and EPC specific markers (CD34, VEGFR2) (Figure 2(a)). The TEM results showed that there was no difference in morphology between sEPC-MVs and aEPC-MVs (Figure 2(b)). With the digestion of RNase, the total RNAs of both EPC-MVs were significantly decreased (Figure 2(c)). More interestingly, the gene expressions of caspase 3 and miR126 were displayed in the same pattern as that in their parent EPCs. The results showed that caspase 3 was upregulated in aEPC-MVs as compared to that in sEPC-MVs or control, and miR126 was down-regulated in aEPC-MVs as compared to that in sEPC-MVs or control (versus control or sEPC-MVs; *P* < 0.05; *n* = 4/group; Figure 2(d)).

### 3.2. The Model of H/R Injury in Hb-ECs Was Confirmed by ROS Overproduction, Cell Dysfunction, and Apoptosis

**Figure 3:**
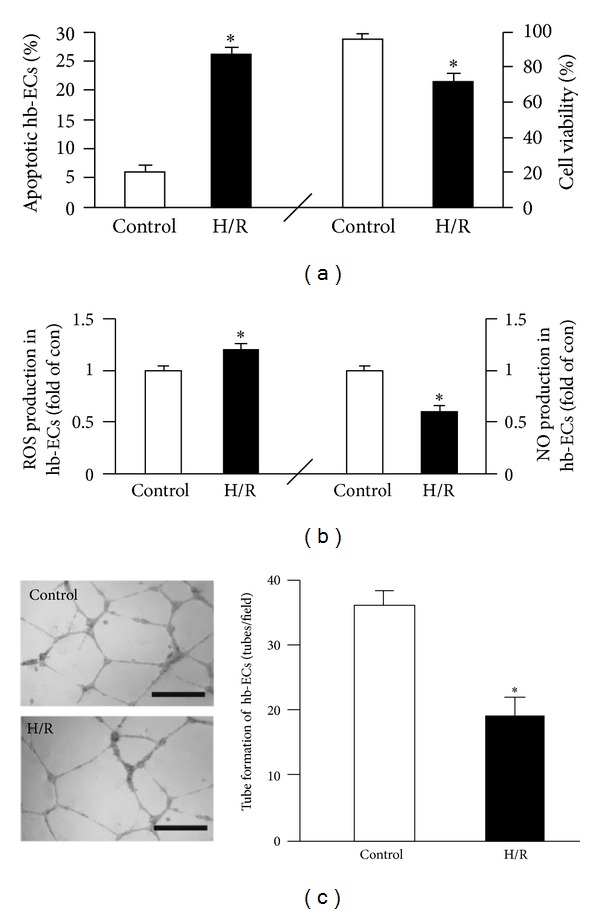
Effects of H/R on hb-EC viability and apoptosis, ROS and NO production, and tube formation. (a) Apoptosis (Annexin V+PI−) and cell viability. (b) ROS and NO production. (c) Tube formation ability. Scale bar: 200 *μ*m. *P < 0.05, versus control; *N* = 4/group.

Hb-ECs were exposed to hypoxia (6 hrs) and followed by reoxygenation (24 hrs). As shown in Figure 3(a), MTT assay showed that H/R decreased hb-EC viability (versus control; *P* < 0.05; *n* = 4/group). To confirm this observation, we also performed apoptotic assay and found that H/R hb-ECs had a higher apoptotic rate than those cultured in normoxia condition (versus control; *P* < 0.05; *n* = 4/group; Figure 3(a)). In addition, in H/R hb-ECs, the ROS production was increased, whereas, the NO production was decreased. And tube formation ability of H/R hb-ECs was decreased (versus control; *P* < 0.05; *n* = 4/group; Figures 3(b)-3(c)). These findings indicated the success of H/R injured model on hb-ECs.

### 3.3. sEPC-MVs Decreased Whereas aEPC-MVs Increased ROS Production and Apoptosis in H/R Hb-ECs via PI3K Pathway

**Figure 4:**
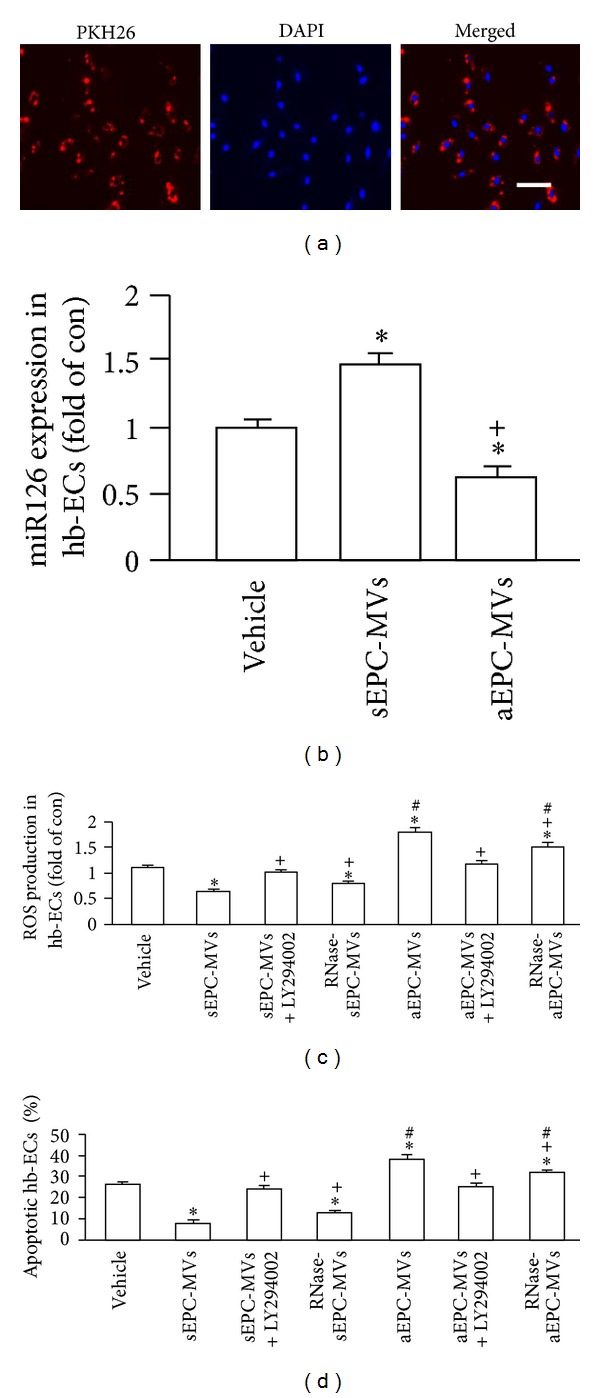
Effects of EPC-MVs on miR126 expression, ROS production, and apoptosis in H/R hb-ECs. (a) Representative images showing the merging of PKH26 labeled EPC-MVs with hb-ECs (red: PKH26; blue: DAPI). Scale bar: 100 *μ*m. (b) miR126 expression in H/R hb-ECs cocultured with aEPC-MVs or sEPC-MVs. (c) ROS production of H/R hb-ECs cocultured with aEPC-MVs or sEPC-MVs. (d) Apoptosis of H/R hb-ECs cocultured with aEPC-MVs or sEPC-MVs. *P < 0.05, versus vehicle; ^+^ *P* < 0.05, versus sEPC-MVs or aEPC-MVs; ^#^ *P* < 0.05, versus sEPC-MVs or RNase-sEPC-MVs; *N* = 4/group.

After coculture the hb-ECs with EPC-MVs for 24 hrs, the PKH26 fluorescence was observed in the cytoplasm of hb-ECs, suggesting the EPC-MVs were incorporated into the hb-ECs (Figure 4(a)). Interestingly, the miR126 expression was upregulated in the hb-ECs cocultured with sEPC-MVs and down-regulated in the hb-ECs cocultured with aEPC-MVs as compared to vehicle (versus vehicle; *P* < 0.05, *n* = 4/group; Figure 4(b)). According to flow cytometric analysis (Figures 4(c)-4(d)), sEPC-MVs decreased the apoptosis level and ROS production in H/R hb-ECs (versus vehicle; *P* < 0.05, *n* = 4/group). On the contrary, aEPC-MVs increased the apoptosis level and ROS production in H/R hb-ECs (versus vehicle; *P* < 0.05, *n* = 4/group). As expected, RNase-sEPC-MVs were less effective on decreasing the level of apoptosis and ROS production in H/R hb-ECs. In addition, preincubation of H/R hb-ECs with PI3K inhibitor (LY294002) abolished the aforementioned effects of sEPC-MVs and aEPC-MVs (*P* < 0.05, *n* = 4/group).

### 3.4. sEPC-MVs Increased Whereas aEPC-MVs Decreased eNOS and NO Production in H/R Hb-ECs via PI3K Pathway

**Figure 5:**
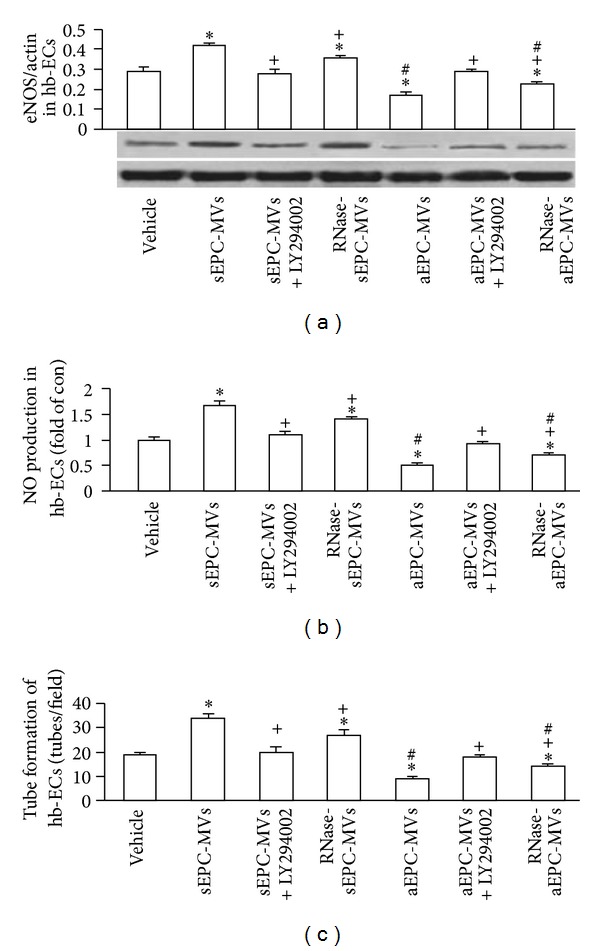
Effects of EPC-MVs on eNOS expression, NO production, and tube formation in H/R hb-ECs. (a) eNOS production of H/R hb-ECs cocultured with aEPC-MVs or sEPC-MVs. (b) NO production of H/R hb-ECs cocultured with aEPC-MVs or sEPC-MVs. (c) Tube formation ability of H/R hb-ECs cocultured with aEPC-MVs or sEPC-MVs. *P < 0.05, versus vehicle; ^+^ *P* < 0.05, versus sEPC-MVs or aEPC-MVs; ^#^ *P* < 0.05, versus sEPC-MVs or RNase-sEPC-MVs; *N* = 4/group.

As shown in Figures 5(a)-5(b), sEPC-MVs significantly upregulated eNOS and NO expressions in the H/R hb-ECs (versus vehicle, *P* < 0.05, *n* = 4/group). On contrary, aEPC-MVs decreased the eNOS and NO production in H/R hb-ECs (versus vehicle; *P* < 0.05, *n* = 4/group). Again, RNase-sEPC-MVs were less effective on increasing whereas RNase-aEPC-MVs were less effective on decreasing the eNOS and NO production in H/R hb-ECs. Similarly, preincubation of H/R hb-ECs with LY294002 attenuated these effects (*P* < 0.05, *n* = 4/group).

### 3.5. sEPC-MVs Increased Whereas aEPC-MVs Decreased the Hb-EC Tube Formation Ability in H/R Hb-ECs via PI3K Pathway

As shown in Figure 5(c), the tube formation ability of H/R hb-ECs was increased after coculture with sEPC-MVs, whereas it was decreased after coculture with the aEPC-MVs (*P* < 0.05, *n* = 4/group). Again, preincubation with LY294002 abolished this effect.

## 4. Discussion

There are three major findings in this study. Firstly, we demonstrated that EPCs stressed with SD (sEPCs) or SD plus TNF*α* (aEPCs) have different changes regarding cell apoptotic rate and the expression levels of caspase 3 and miR126. Secondly, we identified that the MVs released from sEPCs and aEPCs carry the same characters as their parent cells. Thirdly, we confirmed that EPC-MVs can merge with hb-ECs and sEPC-MVs and aEPC-MVs have different functional roles in H/R-induced hb-ECs injury, which might be dependent on their effects on ROS and NO generation, miR126 expression, and regulation of the PI3K/eNOS/NO pathway.

ROS is well known to be implicated in I/R injury [1]. ROS-induced vascular endothelium injury is a pivotal step in the pathogenesis of I/R injury. Although strategies for inhibiting ROS production or increasing their degradation and scavenging have been attempted, they have turned out to be less effective on the prevention or treatment of I/R injury [2]. Therefore, novel approaches are intensely needed for preventing I/R injury.

MVs are cellular fragments, which have been shown to act as a paracrine mediator as they can merge with target cells for exerting their functions [14]. Several studies have demonstrated that MVs released from mesenchymal stem cells can protect the kidney from I/R injury [11–13]. In this study, we generated MVs from EPCs which underwent SD alone or SD plus TNF*α* stimulation and examined the effects of both stimuli on EPCs and their released MVs. We found that both apoptosis and caspase 3 expression of EPCs were significantly increased in the SD plus TNF*α* stimulation group and slightly increased in the SD group. Even more interesting, the miR126, a proangiogenic factor, was upregulated in EPCs stressed by SD and was downregulated in EPCs stressed by SD plus TNF*α*. These data indicate that EPCs underwent different changes in response to different stimuli.

Next, we examined the morphological and functional characteristics of sEPC-MVs and aEPC-MVs. We found that there was no significant difference in morphology and the expression of parent specific markers between sEPC-MVs and aEPC-MVs as determined by TEM and flow cytometry. But there were differences of caspase 3 and miR126 expression between them. According to the ELISA analysis results, the caspase 3 expression in aEPC-MVs was higher than that in sEPC-MVs. The miR126 expression was lower in aEPC-MVs in comparison to sEPC-MVs as revealed by qRT-PCR assay. These data indicate that both aEPC-MVs and sEPC-MVs carry their parent proteins and genetic materials.

Additionally, our data showed that EPC-MVs can be incorporated by hb-ECs, which suggests that EPC-MVs may have a functional role in hb-ECs. In order to further test the potential effects of EPC-MVs, we constructed an H/R hb-EC injury model, which was characterized by overproduction of ROS, an increase in apoptosis, and decrease in NO production, cell viability, and tube formation ability. In coculture experiments, we found that sEPC-MVs protected hb-ECs from H/R-induced ROS overproduction and apoptosis. Both the eNOS and NO production were increased in the H/R hb-ECs cocultured with sEPC-MVs. Moreover, sEPC-MVs rescued the tube formation disability of H/R hb-ECs. In addition, we also observed that these protective effects of sEPC-MVs were abrogated by PI3 K inhibitor LY294002. These data suggest that sEPC-MVs protect H/R hb-ECs from injury via activating PI3K/eNOS/NO pathway. Contrary to sEPC-MVs, aEPC-MVs showed deleterious effects on H/R hb-ECs. Our data showed that aEPC-MVs accelerated ROS overproduction and apoptosis, coupled with decreased eNOS expression and NO generation. Meanwhile, the tube formation ability of H/R hb-ECs was also compromised in the aEPC-MVs groups. Likewise, these deleterious effects of aEPC-MVs were also abolished by LY294002. These findings indicate that aEPC-MVs worsen the oxidative stress in H/R hb-ECs via PI3K/eNOS/NO pathway.

Of note, we also found that all the aforementioned effects of EPC-MVs were partially diminished by coculture with RNase-EPC-MVs. Therefore, it is logical to deduce that EPC-MV carried RNAs which participated in the protective or deleterious effects of EPC-MVs. In the present study, we found that the expression of miR126 in H/R hb-ECs was increased in the sEPC-MVs group, whereas it was decreased in the aEPC-MVs group. On contrary, the caspase 3 expression in H/R hb-ECs was decreased in sEPC-MVs group and increased in aEPC-MVs group. These data suggest that miR126 and caspase 3 were delivered to hb-ECs from EPCs via EPC-MVs, which is in agreement with previous studies showing MVs can deliver and transfer their contents to target cells [8–10]. Nevertheless, the detailed mechanisms of their roles need further investigation.

## 5. Conclusion

Our data demonstrate that sEPCs-MVs and aEPC-MVs are functionally different on H/R-induced apoptosis and dysfunction. These functional roles might rely on the orchestrated mechanisms associated with MV-carried RNAs in control of ROS production and PI3K/eNOS/NO pathway in the target cells. These findings indicate that EPC-MVs could be used as a novel vehicle for treating H/R injury.
